# Orbital-Conjunctival-Eyelid Hemorrhage-Hematoma due to Inappropriate use of FFP2/NK95 Facial Mask in COVID-19

**DOI:** 10.7759/cureus.11273

**Published:** 2020-10-31

**Authors:** Celia Ruiz-Arranz, Enrique Mencía-Gutiérrez, Álvaro Bengoa-González, Silvia Pérez-Trigo, Esperanza Gutiérrez-Díaz

**Affiliations:** 1 Ophthalmology, 12 de Octubre Hospital, Complutense University, Madrid, ESP

**Keywords:** orbit, conjunctival, ffp2/nk95, eyelid, hemorrhage, hematoma, facial mask, covid-19, pandemic

## Abstract

A non-traumatic or spontaneous orbital hemorrhage (NTOH) is usually caused by the presence of an orbital mass, an inflammation, an infection, a bleeding disorder or those called idiopathic. This entity usually affects elderly adults and some risk factors can be identified. The NTOH normally acts like a benign and self-limited process, but attending to its anatomical pattern, may need specific management.

A 64-year-old male referred to us with sudden binocular double vision, without loss of visual acuity (VA) or pain immediately after fitting the FFP2/NK95 facial mask for air protection during the pandemic COVID-19. He presented sudden orbital-subconjunctival-eyelid cutaneous hemorrhage-hematoma with conjunctival protrusion from the palpebral fissure without proptosis. There was also limitation in adduction and a cutaneous hematoma in the inner third of the lower eyelid. After 48 hours, the diplopia had resolved and ocular motility was completely re-established with persistence of a massive hyposphagma. No radiological image test was performed due to the COVID-19 epidemiological situation, as the patient was in good systemic situation and it was not a vital emergency. The evolution was favorable with conservative treatment, cold application, and moisturizing eye drops. After 10 days, the bruising was almost completely gone.

During the ongoing novel coronavirus disease (COVID-19) pandemic caused by the novel enveloped RNA virus named severe acute respiratory syndrome coronavirus 2 (SARS-CoV-2), face mask use has drastically increased among the healthcare professionals and the general population. The importance of this case lies in the new adverse effects caused by the misuse of mandatory face masks in the general population.

## Introduction

During the global pandemic that we suffer due to COVID-19, numerous adverse effects have been described due to the use of facial mask as an individual protective equipment (IPE) especially in healthcare workers (doctors and nurses) [1]. In the study published by Jiang et al., facial wounds consisting of healing damage and pressure ulcers related to the use of facial devices have been described [2]. These same authors report a global prevalence of skin wounds caused by IPE of 42.8% (confidence interval of 95%, 41.3%-44.30%). They differentiate three types of skin damage: pressure, condensation, and sweat. In the regression analysis, they define as additional risk factors, the presence of sweat, daily time of use of IPE, and use of third degree IPE self-filtering masks for particle (FFP3) [1]. The tissue damage produced by IPE manifests itself as a skin wound below the contours of the equipment: sweat, friction, and contact dermatitis.

In the consensus document on safety and prevention of pressure ulcers related to devices it is established that this skin damage is also an additional risk factor for infection, acting as an entryway for COVID-19, other viruses, fungi, and bacteria. They describe a new type of pressure ulcer seen in young, healthy people (healthcare professionals), as well as the prophylactic measures necessary to reduce the incidence of skin damage or injury.

During COVID-19 global pandemic, the demand of masks has been dramatically increased by health professionals and general population. Numerous prestigious institutions, including the World Health Organization, the European Center for Disease Prevention, the Center for Evidence-Based Medicine, and the Ministry of Health of the Government of Spain (MS) have published the features and indications of the different types of masks [3]. Speaking of the general population, the MS has elaborated simple tips for masks use: hand washing before and after use, using correct facial placement covering nose, mouth and chin, avoiding touching them, discarding them if they are damp, and not reusing them if they are not reusable.

All these documents do not collect/include specific adverse effects, and to our knowledge, there is no case report in the scientific literature of an orbital conjunctival-eyelid hemorrhage-hematoma induced by the misuse of IPE in general population.

Subconjunctival hemorrhage is a benign and self-limited process, with a known association with the use of vitamin K antagonists (1.6% incidence in patients receiving warfarin) [4], which generally does not require specific treatment.

On the other hand, a spontaneous orbital hemorrhage is a rare presentation, which is normally caused by an orbital vascular anomaly, an orbital mass, a bleeding disorder, uncontrolled hypertension, or septicemia. The vast majority cause proptosis and diplopia.

It has been reported that a spontaneous retrobulbar hemorrhage is induced by a non-vitamin K antagonist oral anticoagulant (NOAC), rivaroxaban [5]. In contrast, this is a rapidly worsening emergent condition which requires urgent management.

## Case presentation

**Figure 1:**
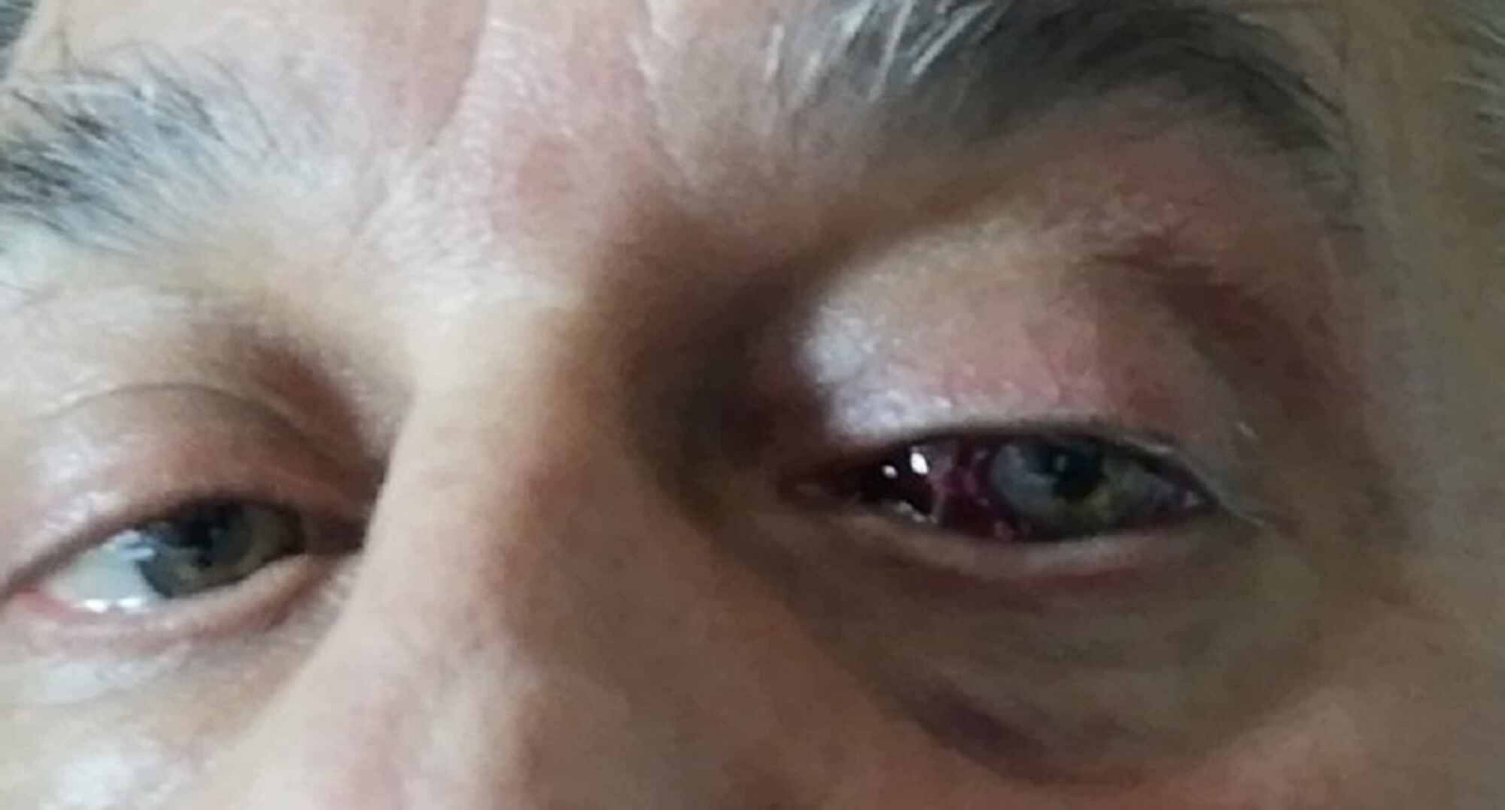
Orbital-conjunctival hemorrhage-hematoma. Orbital-conjunctival hemorrhage-hematoma with secondary left lateral eyeball displacement, and protrusion by eyelid fissure. There was also eyelid ptosis with brow compensation and eyelid crease disappearance.

**Figure 2:**
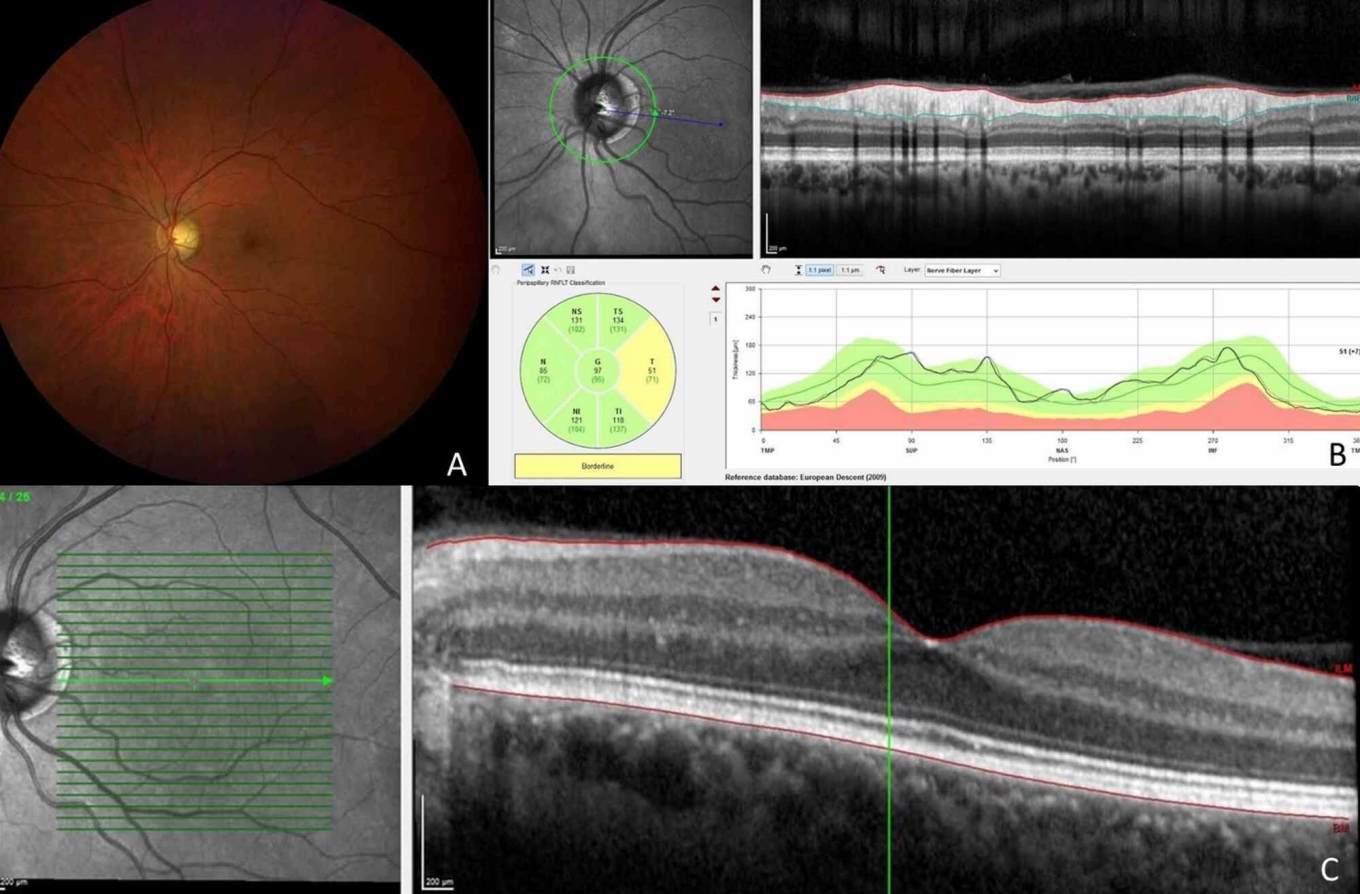
A: Retinography. B: Peripapillary optic nerve fibers optical coherence tomography. C: Macular optical coherence tomography. A: Retinography image was normal. B: Normal optical coherence tomography peripapillary optic nerve fibers layer. C: Macular optical coherence tomography demonstrating a normal retinal image.

A 64-year-old male referred for acute diplopia and a left orbital hemorrhage spontaneously while driving (Figure 1). It happened when he was adjusting a FFP2 mask with the thumb and heart fingers, exerting pressure on the bridge of the nose and inner third of the lower eyelid. He had a medical history of high blood pressure (HBP) and hyperlipidemia, well controlled with medical treatment. No history of tobacco consumption - neither anticoagulant nor antiplatelet therapy. No contact lens user. On examination, there was a massive left subconjunctival hemorrhage and a cutaneous hemorrhage in inner third of the lower eyelid, and restriction of extrinsic ocular motility in adduction of the left eye causing binocular diplopia. There was no proptosis and no pain in ocular movements but the patient had inability to occlude the eyelids due to the left conjunctival hemorrhage. Visual acuity (VA) was preserved and pupillary reflexes were normal without afferent pupillary defect, so no radiological image was performed due to the presence of pandemic, as the patient was in good systemic situation and it was not a vital emergency. Retinography (Figure 2A), optic never fibers layer coherence optic tomography (Figure 2B), and macular optic coherence tomography (Figure 2C) were normal. Arterial blood pressure on arrival, was within normal limits 123/83 mmHg (<135/85). No pathological findings were observed in blood analysis comprising coagulation tests: international normalized ratio (INR) 1.07 s (0.80-1.20), activated partial thromboplastin time (aPTT) 31 s (26-39), prothrombin time (PT) 12.3 s (8.5-13.8), and derived fibrinogen 520 mg/dL (200-560). Screening for inherited thrombophilia was normal.

**Figure 3:**
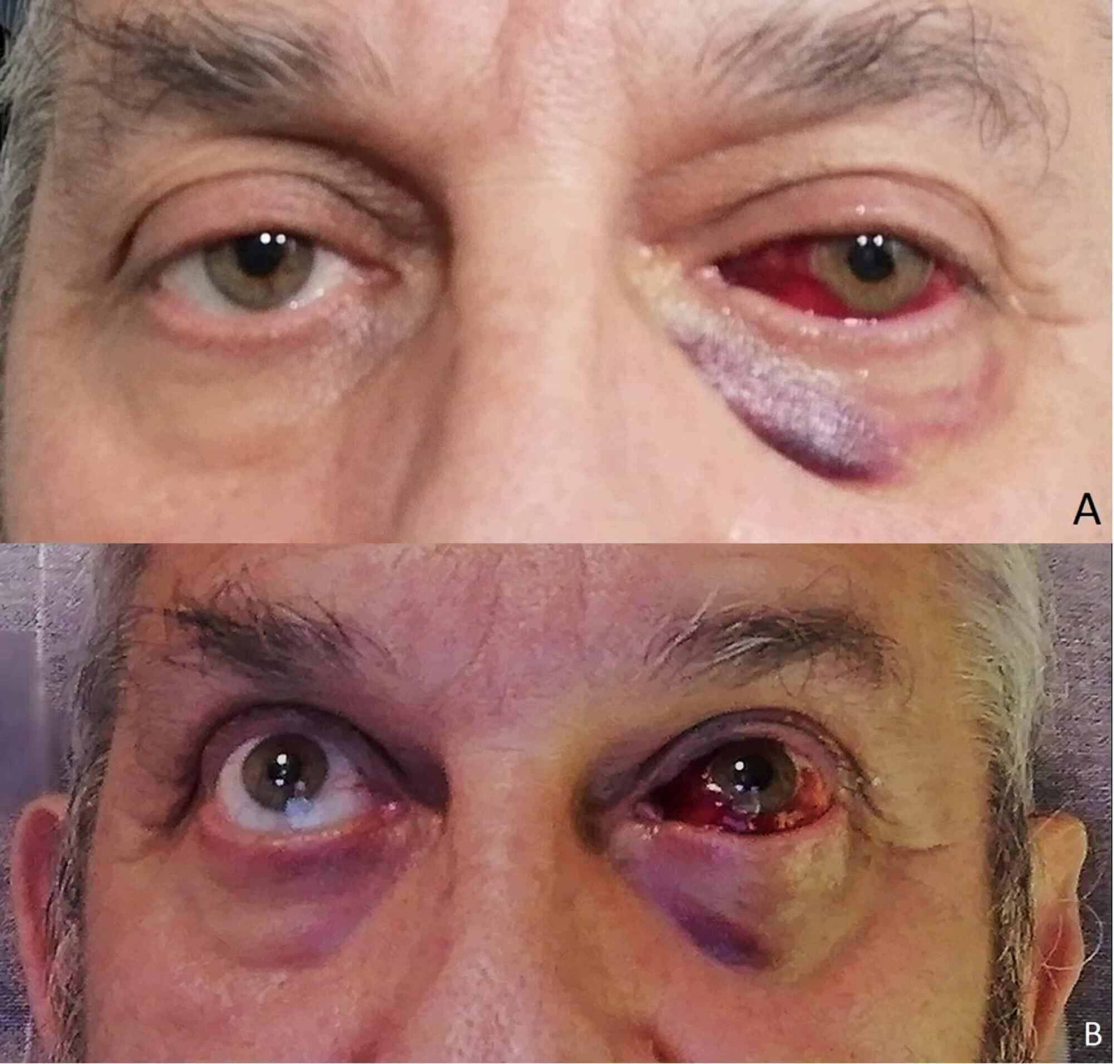
After 48 hours, orbital-conjunctival hemorrhage-hematoma. After 48 hours, orbital-conjunctival hemorrhage-hematoma can still be observed and a localized cutaneous hematoma already in reabsorption can be seen in the inner third of the left lower eyelid corresponding to the place of pressure when adapting the mask.

**Figure 4:**
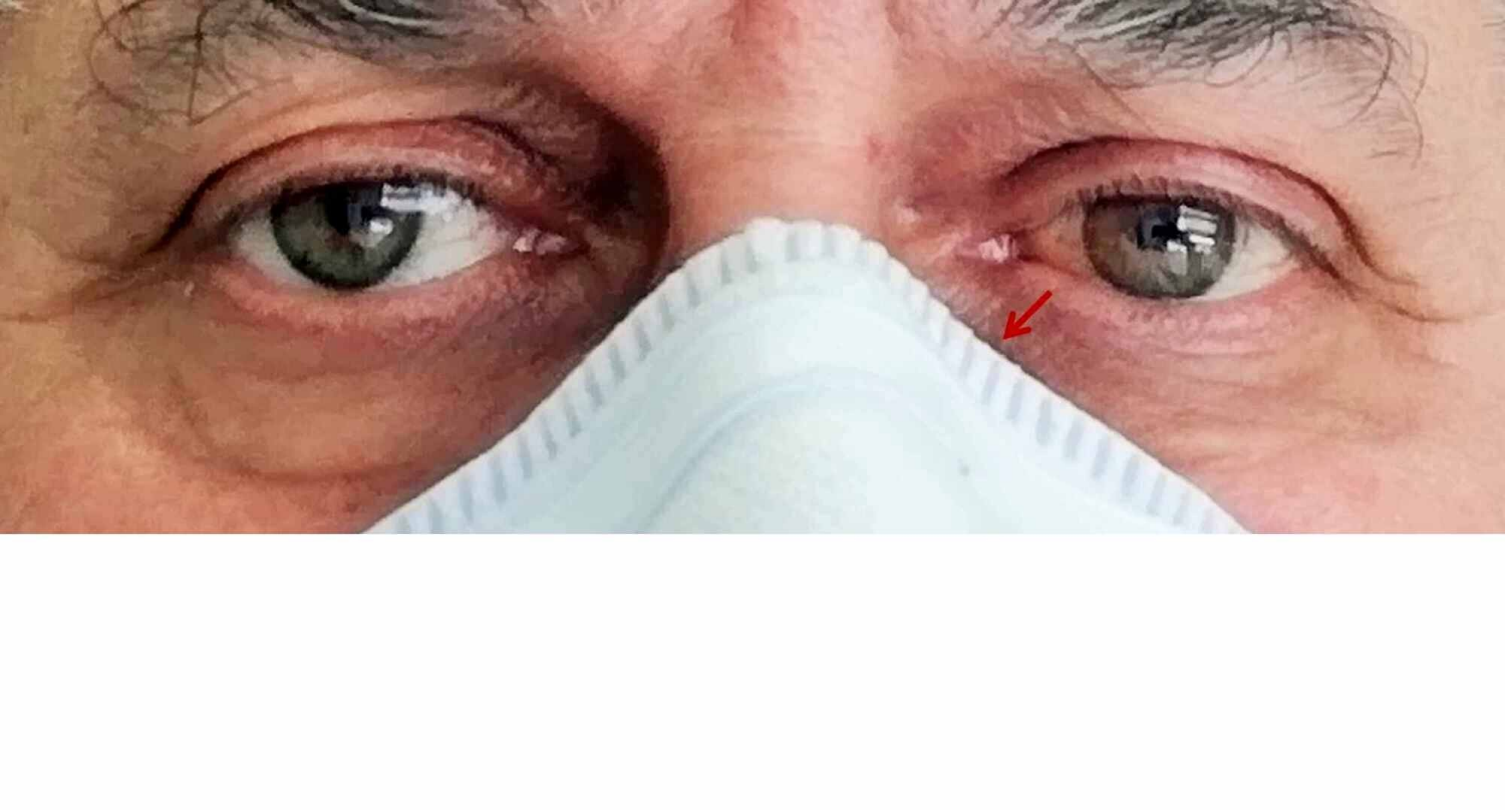
After 10 days, the patient presents normal appearance. After 10 days, the patient presents normal appearance. The arrow indicates the area where pressure was applied and gave rise to the hemorrhage-hematoma on the left side.

Treatment was conservative, insisting on corneal lubrication and application of cold/cold therapy. After 48 hours, the diplopia had completely resolved (Figure 3A-B), and 10 days after the onset, the hematoma was entirely gone (Figure 4).

## Discussion

We can classify orbital hemorrhages into traumatic (TOH) and non-traumatic (NTOH) or spontaneous [5]. The first, which in turn are the most frequent, may be due to local trauma, surgery near the sinuses or orbits, increased cranial venous pressure, barotrauma (diving and even in relation to commercial flights) or after general anesthesia. The NTOH are uncommon and their causes include the presence of orbital masses (vascular abnormalities, benign tumors, or neoplasms), those associated with inflammation, infection (complications of acute sinusitis), those secondary to coagulopathy, and idiopathic cases [6]. In turn, the NTOH can be classified according to the different anatomical patterns that follow: diffuse intraorbital hemorrhage, localized intraorbital hemorrhage (sometimes called a hematic cyst), and bleeding related to orbital floor implants.

One characteristic of the venous system of the orbit is the absence of valves, which explains that an increase in systemic or local pressure can be transmitted directly and produce a symptomatic hemorrhage subconjunctival-orbital. Although it is not possible to establish well-defined risk factors for NTOH, the series published to date conclude that the majority of cases seem to occur in elderly patients, with coexisting cardiovascular risk factors, such as HBP and hyperlipidemia, as well as the use of antiplatelet and anticoagulant agents.

One possible explanation is that these morbidities produce structural damage to the orbital blood vessels, weakening them and predisposing to bleeding. This would be aggravated by the use of antiplatelet and anticoagulants agents, which may increase its extension. Another possible additional factor is that in elderly patients, the nasal septum may be weakened or ruptured, thus communicating the space of the maxillary and ethmoid sinuses with the orbital space and favoring the extension of the hemorrhage.

In the case we present, there was no risk factor for the orbital hemorrhage or were arteriovenous anomaly found.

Among the clinical findings that we can find in the examination, the most frequent is the presence of hemorrhages in ocular tissues such as the skin, the conjunctiva, the vitreous or intraretinal (Purtscher-like retinopathy) [7]. Intraorbital hemorrhage can cause compressive optic neuropathy, with subsequent evolution to optic atrophy if urgent decompression is not performed.

The need to enhance complementary imaging tests should be evaluated according to the etiological mechanism or suspicion, as well as the presence of concomitant neurological symptoms, being the choice intracranial or orbital CT scan.

Initial management is conservative, as long as the VA or optic nerve is not compromised. They usually have a good clinical prognosis with complete resolution in the first weeks, without leaving sequelae.

**Figure 5:**
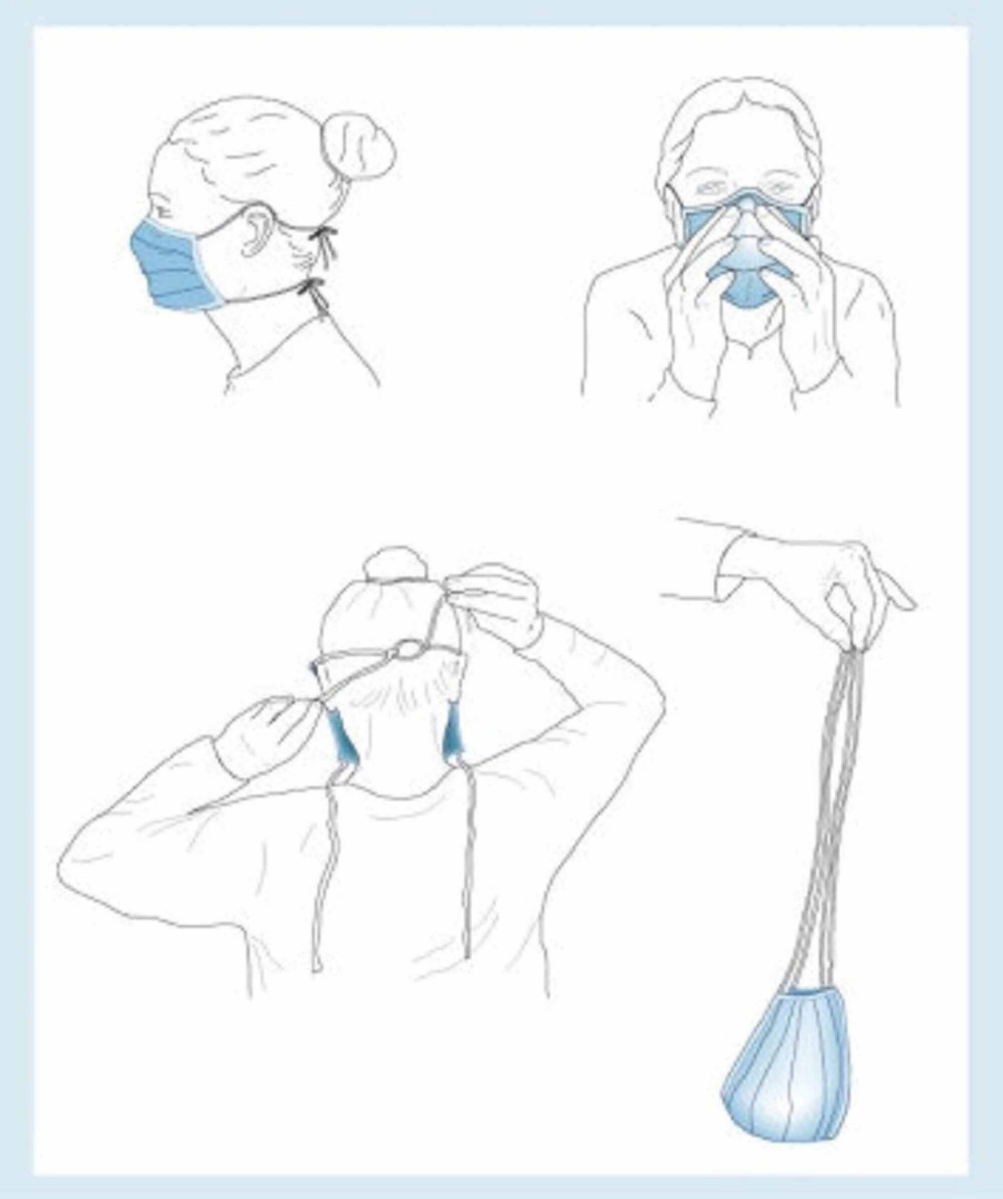
Diagram of correct use of mask. Correct application and removal of face masks [1].

Today there are guidelines for the use of facial protection devices addressed for health professionals, but we cannot forget that during the pandemic COVID-19, the use of these devices has spread to the general population [8]. We therefore report this clinical case, which in the current exceptional situation must be included within the differential diagnosis of all known causes of orbital hemorrhage (diagram of correct use) (Figure 5) [1].

## Conclusions

To date, it has not been reported in the literature any case of secondary conjunctival hemorrhage due to traumatism related to fitting or placement of a facial mask FFP2/NK95. The etiology is not clear, and could have been an accidental rupture of a venous vessel or the possible rupture of an anomalous arteriovenous communication. Treatment was conservative, as there was no suspicion of orbital occupation and possible compression of the optic nerve. The evolution was good with total functional recovery.

It should be emphasized that a mask placement or adjustment should be firm and not forceful in order to avoid unwanted serious complications such as irreversible VA loss, whose incidence may be increased by the widespread use of these devices during the pandemic COVID-19.
